# A Simulation Study of Threats to Validity in Quasi-Experimental Designs: Interrelationship between Design, Measurement, and Analysis

**DOI:** 10.3389/fpsyg.2016.00897

**Published:** 2016-06-16

**Authors:** Fco. P. Holgado-Tello, Salvador Chacón-Moscoso, Susana Sanduvete-Chaves, José A. Pérez-Gil

**Affiliations:** ^1^Metodología de las Ciencias del Comportamiento, Universidad Nacional de Educación a DistanciaMadrid, Spain; ^2^HUM-649, Innoevalua, Psicología Experimental, Universidad de SevillaSevilla, Spain; ^3^Universidad Autónoma de ChileSantiago, Chile

**Keywords:** threats to validity, quasi-experimental designs, Structural Equation Modeling, causal analysis, Classical Test Theory

## Abstract

The Campbellian tradition provides a conceptual framework to assess threats to validity. On the other hand, different models of causal analysis have been developed to control estimation biases in different research designs. However, the link between design features, measurement issues, and concrete impact estimation analyses is weak. In order to provide an empirical solution to this problem, we use Structural Equation Modeling (SEM) as a first approximation to operationalize the analytical implications of threats to validity in quasi-experimental designs. Based on the analogies established between the Classical Test Theory (CTT) and causal analysis, we describe an empirical study based on SEM in which range restriction and statistical power have been simulated in two different models: (1) A multistate model in the control condition (pre-test); and (2) A single-trait-multistate model in the control condition (post-test), adding a new mediator latent exogenous (independent) variable that represents a threat to validity. Results show, empirically, how the differences between both the models could be partially or totally attributed to these threats. Therefore, SEM provides a useful tool to analyze the influence of potential threats to validity.

## Threats To Validity: Theoretical And Analytical Perspectives

The unstable social and political conditions of most contexts to which evaluation programs are applied (Gorard and Cook, 2007) imply that, *a priori*, there are no standardized evaluation design structures (Chacón-Moscoso et al., 2013). Due to this fact and because random assignment of participants to different groups is not always possible (Colli et al., 2014), quasi-experimental designs are more commonly used in social sciences than experimental ones (Shadish et al., 2005). Quasi-experiments lack control over extraneous variables generated by random allocation; therefore, it is extremely important that the evaluation process is conducted in a manner that provides reliable and valid results based on the analysis of the influence of potential threats to validity (Reichardt and Coleman, 1995).

There are conditions other than the intervention program itself that could be responsible for the outcomes. These conditions are called threats to validity which, unless controlled, limit the confidence of causal findings (Weiss, 1998).

This evaluation research context presents two main problems. On the one hand, as a conceptual-theoretical framework, the Campbellian tradition presents a series of threats to validity that can affect four different kinds of validity (Campbell, 1957; Campbell and Stanley, 1963; Cook and Campbell, 1979; Shadish et al., 2002): (a) statistical conclusion validity (García-Pérez, 2012) can be affected by a low statistical power (Tressoldi and Giofré, 2015) and a restricted range (Vaci et al., 2014); (b) internal validity can be affected by selection, history, maturation, and regression; (c) construct validity can be affected by construct confounding, treatment-sensitive factorial structure, and inadequate explication of constructs; and (d) external validity can be affected by interaction of the causal relationship with units or outcomes. Although Campbell’s approach provides a conceptual framework for evaluating the main threats to four types of validity (Shadish et al., 2002) and some guidelines (design features) to enhance validity were presented, there is not an empirical, systematic approach to check and control the influence of threats to validity on the treatment effect estimations in program evaluation practice (e.g., Stocké, 2007; Krause, 2009; Johnson et al., 2015).

On the other hand, from an analytical point of view, procedures have been developed to assess some construct validity threats, such as inadequate explication of constructs, confounding of constructs operations, mono-operationalization, and mono-method bias. Some of these procedures include the multimethod-multitrait approach (Eid et al., 2008) and factor retention analysis, through the study of systematic pattern in the error covariance (Brown, 2015). The apparently useful analytical proposal weakens because it is not based on any theoretical framework.

In sum, there is a small connection between design features, measurement issues, and concrete impact estimation analyses. In order to provide an empirical solution to this problem, we use Structural Equation Modeling (SEM) as a first approximation to operationalize the analytical implications of threats to validity in quasi-experimental designs.

## Structural Equation Modeling (SEM): An Integrated Approach

Based on Steyer (2005), who draws analogies between the Classical Test Theory (CTT)— measurement point of view (Trafimow, 2014) —and causal analysis and Rubin’s Causal Model— design and analysis points of view, which determine the concepts of statistical inference for causal effects in experiments and observational studies (West, 2011) — we assume that SEM can be used to systematize the model assumptions and test the model fit likelihood statistically, and empirically check the way threats to validity affect data and how different threats to validity influence each other.

If we focus on the participant-level scores in each experimental condition, we can establish an analogy between causal analysis and CTT, so that the measurement, design, and analysis aspects would be linked. The participants’ expected value in causal analysis is similar to the true score defined by CTT. That is, it would be the expected score obtained after an infinite number of independent administrations of a measurement, under some assumptions (Lord and Novick, 1968). Based on the concept of parallel test, we can assume that across a set of scores, the variance of the observed score is composed of the sum of the true scores and the error variance. If we consider an experimental or quasi-experimental design with two conditions (control and experimental), then we can expect two true scores for each unit—one for the control condition and another for the experimental condition (Steyer, 2005)—and, therefore, two observed variances (one for the control condition and another for the experimental condition), and one covariance (control-experimental).

Taking into account the number of groups, and the number of measurement occasions, this theoretical framework could be translated into any experimental or quasi-experimental design. For example, if we combine the measurement occasion [pre-test (f0) and post-test (f1)] and the expected value or true score of each group (control: *X* = 0 and experimental: *X* = 1), **Table 1** presents the variance/covariance matrix in a non-equivalent control group design.

**Table 1:** Implied variance/covariance matrix in a non-equivalent control group design.

		Pre (f0)		Post (f1)	
		Control (X0)	Exptal.(X1)	Control (X0)	Exptal. (X1)
Pre (f0)	Control (X0)	***S*^2^ [f0/*X* = 0]**			
	Exptal. (X1)	*S* [f0, f0/*X* = 1, *X* = 0]	***S*^2^ [f0/X = 1]**		
Post (f1)	Control (X0)	*S* [f1, f0/*X* = 0]	*S* [f0, f1/*X* = 0, *X* = 1]	***S*^2^ [f1/*X* = 0]**	
	Exptal. (X1)	*S* [f1, f0/*X* = 1, *X* = 0]	*S* [f1, f0/*X* = 1]	*S* [f1, f1/*X* = 1, *X* = 0]	***S*^2^ [f1/*X* = 1]**

*Pre (f0), pre-treatment time point; Post (f1), post-treatment time point; Control (X0), Control group; Exptal. (X1), Experimental group; S^2^, variance (in boldface); and S, covariance.*

Variances are in the diagonal in boldface; e.g., S^2^[f0/*X* = 0] is the control group variance for the pre-test measurement and S^2^[f1/*X* = 1] is the experimental group variance for the post-test measurement. Covariances are out of the diagonal; e.g., S[f0,f0/*X* = 1, *X* = 0] is the covariance between the control and experimental groups at pre-test; and S[f1,f0/*X* = 0] is the pre-testpost-test covariance for the control group.

From the implied variance-covariance matrix, we can establish the model derivations for the non-equivalent control group design: (a) the control-experimental group covariance in the pre-test (S[f0,f0/*X* = 1, *X* = 0]) should be equal to the control and experimental variance in the pre-test (S^2^[f0/*X* = 0]; and S^2^[f0/*X* = 1]); equal to the control variance in the post-test (S^2^[f1/*X* = 0]); and equal to the pre-testpost-test control group covariance S[f1,f0/*X* = 0]); and (b) the pre-testpost-test experimental group covariance (S[f1,f0/*X* = 1]) should be equal to the control-experimental group covariance in the post-test (S[f1,f1/*X* = 0, *X* = 1]); and equal to the pretestcontrol posttest-experimental covariance (S[f1,f0/*X* = 1, *X* = 0]).

**FIGURE 1:**
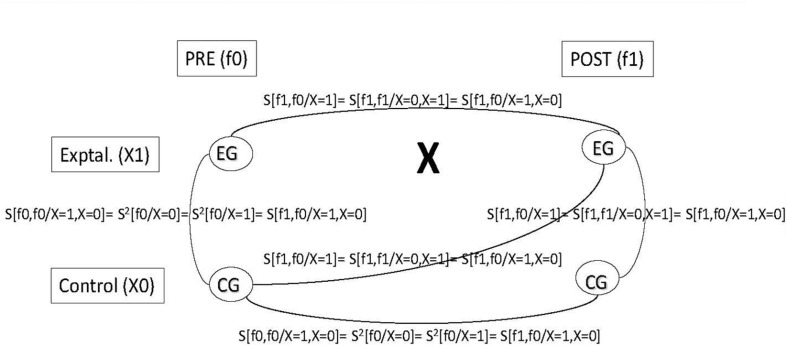
**Covariances in a non- equivalent control group design.** Pre (f0), pre-treatment measurement occasion; Post (fl), post-treatment measurement occasion; Control (X0), Control group; Exptal. (XI), Experimental group; *S^2^*, variance; *S*, covariance.

These assumptions are shown in **Figure 1** over a non-equivalent control group design scheme.

The variance-covariance derivations could be operationalized via SEM through restriction of models, including more latent variables or testing the error terms, and therefore statistically tested. For example, the true scores (expected values or μ, the means of the population) of the control and experimental groups should be significantly equivalent in the pre-test (f0) and significantly different in the post-test (f1); in the control group, true scores should be significantly equivalent between the pre- (f0) and post-test (f1); and in the experimental group, these expected values should be significantly different between the pre- (f0) and post-test (f1). We can design these restrictions via SEM, whether working with one or more groups or measurement occasions (Bollen and Curran, 2006). If the above conditions are not satisfied, it may be due to any validity threats that could be tested in an SEM framework. At this point, it is important to emphasize that this approach is only applicable in cases when the intervention aims to change the level of the dependent variable/s. However, when the aim is to maintain it, then the logic would be different (the expected changes would be in the control group across the pre-test and the post-test, rather than in the experimental group).

**FIGURE 2:**
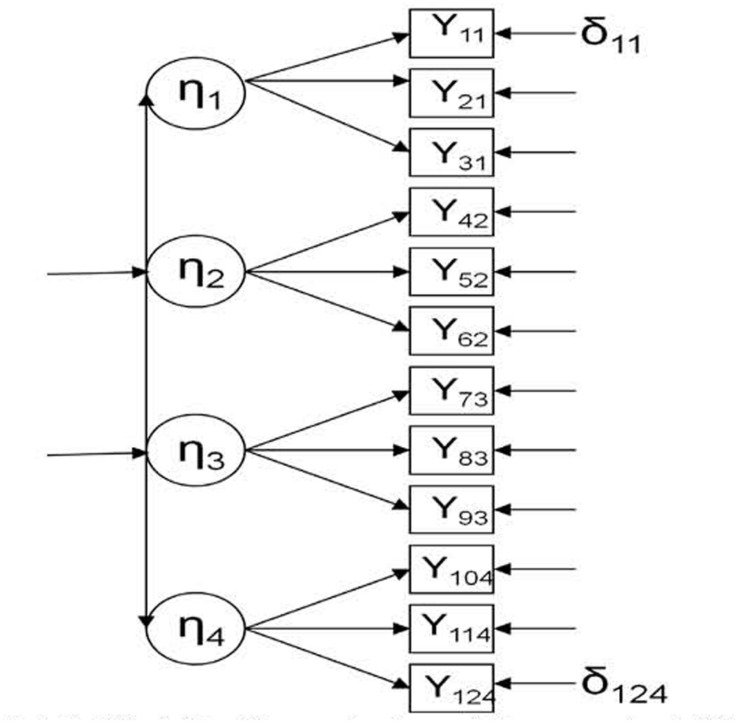
**Multistate model in the control condition, pre-test (Model 1).** η, latent endogenous (dependent) variable; *Y*, observed endogenous (dependent) variable; δ, error.

Once we have established the theoretical assumptions, the next step is to try to draw a non-equivalent control group into the SEM framework. As an example, we opt to use only one group (control group) in a simple design (pre-test and post-test) because the conditions are more easily simulated (a more complex design and model with control and experimental groups would require two pre-tests and two post-tests). In this sense, **Figure 2** presents a multistate model where four different endogenous latent variables are measured at the same time (in the pre-test).

Believable inferences are based on the assumption that all changes between the pre-test and post-test are caused by the treatment, and this assumption can only be true if we do not find systematic changes between the pre-test and post-test in the control group.

**FIGURE 3:**
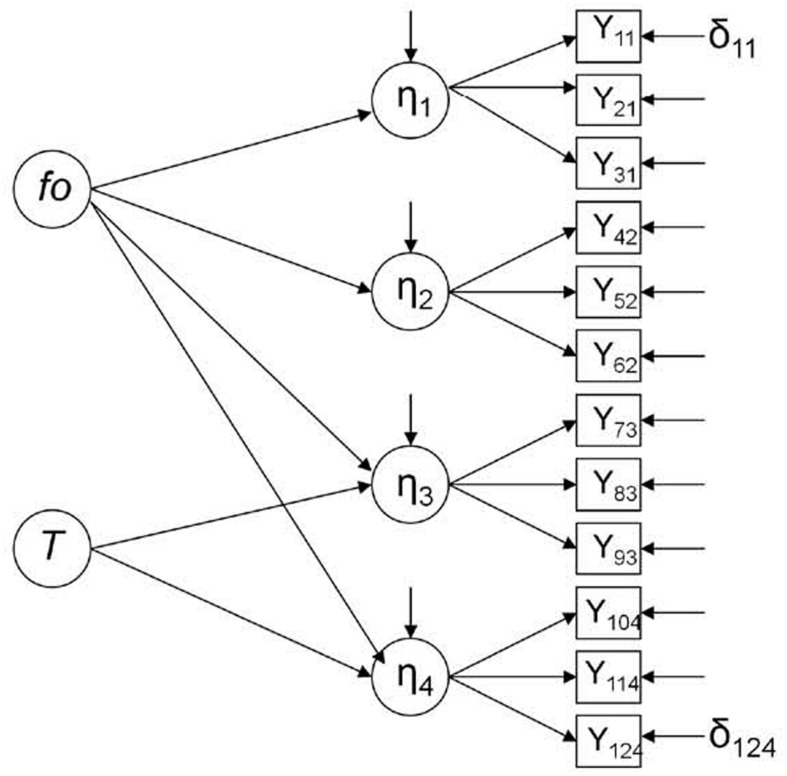
**Singletrait-multistate Model in the control condition, post-test (Model 2; Steyer, 2005).** *fo*, latent exogenous (independent) variable representing the expected outcome under control condition; *T*, latent exogenous (independent) variable representing a threat to validity; η, latent endogenous (dependent) variable; *Y*, observed endogenous (dependent) variable; and δ, error.

Then, we can suppose that *X* = 1 in the variance-covariance matrix is not an experimental group (i.e., a group that participated in a treatment), but a group affected by a threat to validity that can modify the data and generate systematic changes. In a parallel way, the latent variable *T* represented in **Figure 3** is not a treatment, but a threat to validity, so this figure would represent the control group in a concrete time point (the post-test), where an odd element, such as history, for example, can be affecting the results in two of the four endogenous latent variables, i.e., η_3_ and η_4._ Let’s suppose that, to measure the effectiveness of an intervention program to improve attitudes toward immigration in a developed country with an aging population, participants from an experimental group and a control group filled in a questionnaire at an early stage (pre-test). A year later, after the implementation of the intervention program, the post-test was completed. This questionnaire was formed by four dimensions: public safety (η_1_), education (η_2_), economy (η_3_) and public health (η_4_). It was not expected to obtain significant differences between pre- and post-test measures in the control group. However, a wave of young immigrants (*T*) occurred concurrently with the study and, according to research, promoted an increase in economic activity and an improvement in public health by increasing the number of taxpayers. Thus, in the control group, there was a significant improvement in attitudes toward immigration in economy (η_3_) and public health (η_4_), while attitudes in public safety (η_1_) and education (η_2_) did not vary significantly.

At this point, it is important to clarify that this is just a hypothetical situation; the model could have been defined with the possible influence of *T* over three endogenous latent variables instead of two, over only one, over η_2_ and η_3_ instead of over η_3_ and η_4_, and so on.

In this case, we expect the same results in all the variances and covariances presented in **Table 1.** As **Figures 2** and **3** represent, respectively, pre- and post-test in the control group, any systematic change found could be attributed to the influence of a threat to validity (*T*).

## Advantages Of SEM Over Other Methods

Scarce research in psychology was aimed to empirically detect the influence of threats to validity in interventions based on a theoretical framework. In this regard, Crutzen et al. (2015) used meta-analysis in order to study the relationship between differential attrition and several moderator variables; nevertheless, they could not study the relationship between the differential attrition and the effect size owing to technical problems. Furthermore, Damen et al. (2015) carried out a longitudinal study to finally conclude that a possible attrition bias occurred in a percutaneous coronary intervention, as drop-outs and completers differed systematically on some socio-demographic, clinical, and psychological baseline characteristics; nevertheless, as drop-outs did not receive the complete intervention, the authors could not study the difference across both groups (drop-outs and completers) in the results obtained in the post-test. Mixed-effects regression is useful to study the difference between the pre- and post-test across experimental and control groups. Nevertheless, this option is based on a pure analytical perspective and is restricted to include only directly observed variables; whereas, SEM is not just based on analysis, but on the integration of design, measurement, and analysis. Thus, it provides the possibility of obtaining concrete data about the relationship between latent and observed variables used to measure them and the associated measurement error for each one (measurement model), and the relationship between different latent variables (structural model), as shown in Duncan et al. (2006). Additionally, when the design presented includes two groups, the degree of equivalence between them can be defined depending on the restrictions imposed: we can assume equivalence between experimental and control groups in both the measurement and the structural model, or only in one of them. As a consequence of these differences, regression tends to obtain less sensitive results compared with SEM (Nusair and Hua, 2010).

Therefore, the SEM framework presents several advantages compared with other procedures. This approach includes a measurement-design-analysis point of view, so it is more complete than the traditional approaches based on a single aspect. Moreover, it allows to (a) take conclusions about the relationship across multiple latent variables between them (structural model) and each latent variable with the observed variables that measure it (measurement model); (b) define the degree of equivalence between the experimental and the control group; and (c) obtain inferences about the influence of threats to validity in the results; (d) be generalized to any threat to any kind of validity; and (e) study the concrete conditions under which different threats to validity could be influencing the results.

A similar methodology has been already found as useful to study the influence of threats to validity in other fields; e.g., (a) in forest research, Ficko and Boncina (2014) operationalized the influence of response style bias and the robustness of statistical methods in the results using simulations and including latent variables in the models representing those threats to validity; and (b) in medical research, Mickenautsch et al. (2014) studied the inflation of effect size owing to selection bias using simulations. In the current study, we show the application and usefulness of simulations and the SEM framework in social sciences, specifically in psychology, to detect the influence of other different threats to validity.

## Objective

The objective of this study is to illustrate conceptual problems of threats to validity through causal analyses using SEM, under the framework of design. Concretely, based on the multistate and single-trait-multistate models, we carry out a simulation study where two threats to statistical conclusion validity are manipulated (restriction of range and low statistical power) in order to analyze the way in which a third threat to validity named *T* (unspecified, it could be potentially any of them) could be affecting the measures in the post-test of a non-equivalent control group in a quasi-experimental design.

## Materials and Methods

Data was generated using two different models. However, in both the cases, we considered only the control condition: (a) **Figure 2** represents the multistate model in the control condition (Model 1), where four latent endogenous (dependent) variables (η) are measured through three observed endogenous variables (*Y*) in a concrete time point (pre-test); (b) **Figure 3** represents the single-trait-multistate model in the control condition (Model 2), where the same four latent endogenous variables are measured through the same three observed endogenous variables in another concrete time point (post-test) (Steyer, 2005; Dumenci and Windle, 2010; Pohl and Steyer, 2010). In this case, a new mediator latent exogenous (independent) variable that represents a threat to validity (*T*) was added in order to detect its possible influence in the model fit; *f_0_* is another latent exogenous variable that represents the expected outcome under the control condition (Steyer, 2005). Both Models 1 and 2 assume that all effects are linear (Kline, 2012).

When the multistate model (Model 1, pre-test in control group; **Figure 2** that does not include *T*) is rejected and the single-trait-multistate model (Model 2, post-test in control group; **Figure 3** that includes *T*) is accepted, we can conclude that the *T* variable could be affecting the data in the post-test; thus, differences found between the pre-test and the post-test could be partially or totally attributed to threats to validity. Under these circumstances, further analysis would not provide valid inferences about the effectiveness of treatment. In that case, the *T* variable could be operationalized in a SEM (Ryu, 2014).

Additionally, two previously mentioned threats to statistical conclusion validity are manipulated in order to study the possible interaction with the threat to validity named *T* in **Figure 3**: (a) the *low statistical power* implies obtaining non-significant relationship between the treatment and outcome because the experiment has insufficient power (probability of finding an effect when the effect exists). This threat to validity was manipulated by varying the sample size, with 5 conditions: 100, 500, 750, 1000, and 5000 participants; and (b) the *restricted range* implies that reduced range on a variable usually weakens the relationship between this variable and another (Coenders and Saris, 1995; DiStefano, 2002; Holgado-Tello et al., 2010; Yang-Wallentin et al., 2010; Williams and Vogt, 2011; Bollen, 2014). This threat to validity was manipulated by varying the number of levels in the dependent observed variables (*Y*), with four conditions: 3, 5, and 7 discrete categories, and as continuous variables.

For each latent endogenous variable, three observed variables were simulated. The factor loadings of the observed variables were always the same in all factors (0.8). The simulated factor loadings were high in order to avoid doubts about the specification in the estimation stage of the parameters. Observed variables were generated according to a normal distribution *N(0,1)*. Then, these answers were categorized according to 3, 5, and 7 discrete categories, that is, were categorized in Likert scales with different numbers of possible responses to restrict the range of variation, or remained as continuous variables. The responses to all observed variables remained symmetrical in order to avoid the influence of skewness. To categorize the Likert scales, as stated by Bollen and Barb (1981), the continuum was divided into equal intervals from *z* = –3 to *z* = 3 in order to calculate the thresholds of the condition in which the response distribution to all items is symmetrical (skewness = 0). Finally, the sample size had five experimental values (100, 500, 750, 1000, and 5000).

The combination of the two experimental factors produced 20 experimental conditions (4 × 5) which were replicated 1000 times. These replications were performed using R version 2.0.0, which invoked PRELIS successively (Jöreskog and Sörbom, 1996b) to generate the corresponding data matrices according to the specifications resulting from the combination of the experimental conditions. Thus, for each data generated matrix, correlation matrices were obtained. After obtaining correlation matrices for each replication, the corresponding Confirmatory Factor Analysis was performed successively. The instrumental problem of underidentification in Model 2 (**Figure 3**) was solved by constraining four model components as equal to one: two beta parameters (concretely, β_11_ and β_32_) and the variances of *F_0_* and *T*.

As in the previous case, these replications were performed using R version 2.0.0, which invoked LISREL 8.8 successively (Jöreskog and Sörbom, 1996a).

## Results

**Table 2** presents the results obtained in the multistate model in the control condition, pre-test (Model 1) and the single-trait-multistate model in the control condition, post-test (Model 2) in the different experimental conditions.

**Table 2:** Results obtained in Models 1 and 2 in different conditions.

*n*	Categories	% Accepted Ho		% Λχ^2^ is significant (Λ*df* = 3)	% *RMSEA* < 0.08	
		Model 1	Model 2		Model 1	Model 2
**100**	**3**	0.3	0.3	**72.6**	0.4	1.3
	**5**	0.0	0.9	**100**	0.9	17
	**7**	0.0	0.0	**99.9**	0.0	1.5
	**Con.**	0.0	0.0	**99.1**	1.7	7.5
**500**	**3**	0.0	0.0	**100**	0.0	29
	**5**	0.0	5.4	**100**	**4.1**	**100**
	**7**	0.0	0.0	**100**	**0.1**	**94.7**
	**Con.**	**3.5**	**95.6**	**100**	100	100
**750**	**3**	0.0	0.0	**100**	**0.0**	**84.5**
	**5**	0.0	6.7	**100**	**4.0**	**100**
	**7**	0.0	0.1	**100**	**0.4**	**99.8**
	**Con.**	**0.9**	**96.4**	**100**	100	100
**1000**	**3**	0.0	0.0	**100**	**0.2**	**99.3**
	**5**	0.0	6.3	**100**	**4.7**	**100**
	**7**	0.0	0.0	**100**	**0.6**	**100**
	**Con.**	**0.0**	**93.9**	**100**	100	100
**5000**	**3**	0.0	0.0	**100**	**0.0**	**99.9**
	**5**	0.0	6.5	**100**	**0.6**	**100**
	**7**	0.0	0.1	**100**	**0.4**	**100**
	**Con.**	**0.0**	**96.4**	**100**	100	100

*Model 1, the pre-test in control group, which does not include a possible threat to validity influence (T); Model 2, the post-test in control group, which includes a possible threat to validity influence (T); n, sample size; % accepted Ho, percentage of null hypothesis accepted (i.e., the model fits) in Models 1 and 2 using χ^2^; % Λχ^2^ is significant, percentage of significant increase in χ^2^ between Models 1 and 2; Λdf, increase in the degrees of freedom between Models 1 and 2; % RMSEA < 0.08, percentage of occasions in which the Root Mean Square Error of Approximation is under 0.08 (i.e., the model fits); Con., the dependent variables (Y) are continuous. Values marked in bold are the results that suggest a better fit in Model 2 than in Model 1.*

We found, in general, that: (a) in none of occasions Model 1 fitted better than Model 2; (b) increase in chi-square (Λχ^2^) was significant from Model 1 to 2; therefore, Model 2 fitted significantly better than Model 1 in all the experimental conditions in most of the replications (in 100% of replications when there were 500 participants or more); (c) with 100 participants, both models were rejected, regardless of the categorization of the observed dependent variables (*Y*).

Taking into account the percentage of replications where *RMSEA* was lower than 0.08 we found the following results: (a) with 500 participants or more, both Models 1 and 2 fitted when the observed dependent variables (*Y*) were continuous; and (b) Model 2 fitted better than Model 1 when the observed dependent variables (*Y*) had 5 or 7 categories; with 750 participants or more as well, the same result was found in the case that the observed dependent variables (*Y*) had 3 categories.

Taking into account the percentage of accepted null hypothesis considering χ^2^, an index more sensible than *RMSEA*, the only model that fitted was Model 2 in the case where there were 500 participants or more and the observed dependent variables (*Y*) were continuous.

Whether the multistate model (Model 1) is rejected and the single-trait-multistate model (Model 2) is accepted, following the results obtained, *T* could be affecting the results: (a) in all the experimental conditions, if we consider the percentage of significant increase of chi squared values (% Λχ^2^); (b) when there were 500 participants or more and the observed dependent variables (*Y*) had 5 or 7 categories, if we consider the percentage of *RMSEA* lower than 0.08 (% RMSEA < 0.08); and (c) when there were 500 participants or more and the observed dependent variables (*Y*) were continuous, if we consider the percentage of accepted null hypothesis considering χ^2^.

Following the same logic, we can conclude that the possible effect of the threat to validity (*T*) was only annulled in the case that we had at least 500 participants and the observed dependent variables (*Y*) were continuous, if we consider the percentage of *RMSEA* lower than 0.08 (% RMSEA < 0.08).

## Discussion

We would like to remark that the current study is a very preliminary approximation to study the threats to validity in quasi-experimental designs under the Campbellian tradition. We have attempted to present the basic aspects of the conceptual foundations to approach the study of threats to validity from an empirical perspective. The combination of design, CTT, and SEM has enabled us to obtain the design models derivations expressed in a variance-covariance matrix whose likelihood could be tested via SEM. Finally, from a pragmatic perspective, we have attempted to empirically illustrate the proposals presented via a simulation study. This study is an attempt to open slightly a door to develop vast empirical research for the solution of the problem regarding the threats to validity. From this perspective, we suggest potential future research to analyze other types of validity taking into account many possible designs.

Overall, we conclude that the single-trait-multistate model in the control condition, post-test (Model 2, including *T*), presented a better fit than the multistate model in the control condition, pre-test (Model 1, without *T*), across the experimental conditions. As the number of categories and sample size increase, the results showed that Model 1 was rejected in favor of Model 2.

The key findings obtained based on the simulation study of threats to validity using SEM applied to causal analysis are as follows: (a) a general view including measurement, design, and analysis aspects can be provided, bridging design issues and analytical implications, by analytically studying the consequences of threats to validity; this would give a necessary insight for practitioners when considering the consequences of design features on impact analysis; (b) it is useful to include several variables in the analysis using SEM representing any threat to any kind of validity in order to explain the inter-individual differences in the individual causal effects of the treatment variable on the response variable; with SEM, a test of measurement invariance using a concrete set of data could be carried out in a complementary way to study the possible differences between models, obtaining conclusions for specific situations in specific conditions (Muthén and Asparouhov, 2013); the advantage of using simulations is that conclusions about possible threats to validity can be easily generalized to any situation and to different conditions due to the high number of replications obtained (1000 in this study) and the possibility of manipulating different variables (e.g., number of possible responses and sample size in this study); thus, based on this study, we can conclude that (c) it is recommended not to categorize the dependent variables and, when done, try to have as many categories as possible; with continuous dependent variables, the possible negative effect of the threat to validity included in Model 2 (named *T*) tended to be neutralized (Model 1, without *T*, also obtained a good fit considering *RMSEA*); and (d) using small sample sizes (less than 100 participants) is not adequate (Models, including *T* or not, did not present an acceptable fit).

For future research: (a) we shall apply the procedure presented in the current study using real data obtained from a real situation in order to show practitioners how this proposal can increase the control over extraneous variables and, consequently, the quality of the interventions; (b) it will be necessary to work under the logic of multigroup analysis. This perspective would enable us to consider the control and experimental groups at the same time, and the pre- and post-test measures; then, it would be possible to impose the restrictions of the variance-covariance matrix of **Table 1.** In this way, some weaknesses of the present proposal would be solved, such as the fact that extraneous variables do not necessarily imply a threat to validity because, when provoking the same effect in the treatment and control groups, this effect is neutralized (for example, the effect of maturation in children); in this sense, we would find a positive change in the control group when comparing pre- and post-test (instead of the same true score), but the change would be significantly lower than in the treatment group (if the treatment were effective). In sum, the control group does not need to have an identical level of X in pre and post-test, but this possible level difference does not need to be statistically significant compared to the treatment group. These differences can only be detected when comparing both groups (control and experimental) and both measurement occasions (pre and post-test); (c) when working with control and experimental groups, we shall manipulate the degree of equivalence between both to study the changes in the model fit when control and experimental groups are strictly equivalents (strong equivalence; i.e., equal structural and measurement model), or only the structural model is equal between control and experimental groups, or only the measurement model is equal between control and experimental groups (weak equivalence). Thus, it has completely different consequences on possible inferences to be made from the quasi-experimental designs. If a strong equivalence is achieved, then we would have empirical evidence of a “high degree of validity” in the results obtained. However, when the equivalence found is only weak, we could suspect that some threats to construct validity could be affecting the results when the equivalence is found only in the measurement model, and it is possible that some threats to internal validity could be working if the equivalence is achieved only in the structural model; and (d) we shall manipulate other threats to validity (Shadish et al., 2002) using the same approach; e.g., violated assumptions of statistical tests (a threat to statistical conclusion validity) can be studied by simulating data with and without normal distribution and checking if the same model fits under both conditions; regression (a threat to internal validity) can be studied by simulating data sets with and without extreme values and checking if the same model fits under both conditions; treatment-sensitive factorial structure (a threat to construct validity) can be studied by simulating possible changes in data when comparing pre- and post-test results and checking if the factorial structure obtained in the pre-test is maintained equal in the post-test; inadequate explication of constructs (another threat to construct validity) can be studied by taking real data obtained from questionnaires and, before checking the possible relationships between constructs (structural model), confirming that items measure adequately each construct (measurement model); or interaction of the causal relationship with units (a threat to external validity) can be studied by checking the measurement invariance of a model across different groups, such as male and female.

## Author Contributions

FH-T and SC-M came up with the initial idea and design of the work and interpreted the results. FH-T, SC-M, and JP-G made critical revisions to the manuscript for important intellectual content. FH-T and JP–G performed the analyses. SS-C helped out in the interpretation of data and was in charge of drafting the manuscript. All the four authors (FH-T, SC-M, SS-C, and JP-G) provided the final approval of the version to be published, and agreed to be accountable for all aspects of the work in ensuring that questions related to the accuracy or integrity of any part of the work are appropriately investigated and resolved.

## Conflict of Interest Statement

The authors declare that the research was conducted in the absence of any commercial or financial relationships that could be construed as a potential conflict of interest.
